# An LLM-based decision aid for collaborative decision-making in juvenile idiopathic arthritis: A multimethod evaluation

**DOI:** 10.1371/journal.pdig.0001655

**Published:** 2026-10-22

**Authors:** Antonia Barbaric, Susanne M. Benseler, Chitra Lalloo, Xinyi Celine Liu, Alex Mariakakis, Quynh Pham, Sebastiaan J. Vastert, Rae S. M. Yeung, Joseph A. Cafazzo, Joost F. Swart

**Affiliations:** 1 Institute of Health Policy, Management, and Evaluation, University of Toronto, Toronto, Ontario, Canada; 2 Centre for Digital Therapeutics, University Health Network, Toronto, Ontario, Canada; 3 Department of Pediatrics, Cumming School of Medicine, University of Calgary, Calgary, Albereta, Canada; 4 Children’s Health Ireland, and University College Dublin, Trinity College Dublin, Dublin, Ireland; 5 Child Health Evaluative Sciences, Research Institute, The Hospital for Sick Children, Toronto, Ontario, Canada; 6 Department of Computer Science, University of Toronto, Toronto, Ontario, Canada; 7 Telfer School of Management, University of Ottawa, Ottawa, Ontario, Canada; 8 Department of Pediatric Rheumatology and Immunology, Wilhelmina, Children’s Hospital, University Medical Center Utrecht, Utrecht, The Netherlands; 9 Faculty of Medicine, Utrecht University, Utrecht, The Netherlands; 10 Center for Translational Immunology, University Medical Center Utrecht, Utrecht, The Netherlands; 11 Department of Immunology and Medical Science, University of Toronto, Toronto, Ontario, Canada; 12 Division of Rheumatology, The Hospital for Sick Children, Toronto, Ontario, Canada; Instituto Politécnico Nacional Escuela Superior de Medicina: Instituto Politecnico Nacional Escuela Superior de Medicina, MEXICO

## Abstract

Juvenile idiopathic arthritis (JIA) is the most common pediatric chronic inflammatory rheumatic condition, with substantial quality-of-life impacts. Treatment selection is complex due to limited guideline evidence, and difficult trade-offs. Decisions are often solely physician driven. We evaluated the feasibility of Large Language Model-based Decision Aid (LLM-DA) designed to support collaborative rather than solely physician decision-making in JIA care. A multi-method feasibility study was conducted in accordance with the TRIPOD-LLM checklist. The aims were twofold: first, to assess the quality of the LLM-DA (OpenAI’s o3 model) based on evaluations from three pediatric rheumatologists and an exploratory assessment by an LLM-as-a-Judge (OpenAI’s GPT-5 model); and second, to characterize perceived value of the tool from the patient perspective through a mixed methods evaluation (n = 34). For 10 de-identified use cases, rheumatologists and LLM-Judge generated treatment recommendations and scored the LLM-DA outputs on safety, factual consistency, treatment appropriateness, and preference acknowledgment. Rheumatologists rated 97% of outputs as acceptable for safety and preference acknowledgment, 60% for factual consistency, 70% for appropriateness. Treatment recommendation agreement among all evaluators and real-world data was low at <30%. Inter-rater agreement for scores among rheumatologists was *almost perfect* for safety and preference acknowledgement, *moderate* for treatment appropriateness, and *poor* for factual consistency, and remained the same or improved with LLM-as-a-Judge. Variability in evaluator scoring and treatment recommendations may reflect the inherent heterogeneity in acceptable JIA treatment pathways and potential limitations in the LLM-DA’s decision-making logic. Five patient themes were identified: the tool complements rheumatologist expertise, prepares patients through reflection and actionable information, builds confidence for self-advocacy, has variable value depending on the patient-physician relationship, and has potential for broader JIA self-management. These exploratory findings provide a proof-of-concept for using AI to support collaborative decision-making, while highlighting the need for future studies to determine whether LLMs can reliably support clinical practice.

## Author summary

Our work investigates the feasibility of using large language models (LLMs), to support treatment planning for juvenile idiopathic arthritis (JIA). JIA is hard to treat because evidence in clinical guidelines is low, so physicians often rely on their experiential knowledge to make decisions, and medications can be hard to manage due to harsh side effects. Given these challenges, there is a unique opportunity to investigate whether decision aids that use LLMs can help patients get more involved in their care. Our research provides preliminary evidence that the LLM-DA may support physician-mediated discussions, particularly by helping patients reflect on preferences and prepare for appointments. We also interviewed 34 individuals diagnosed with JIA and found that they thought the decision aid’s outputs were useful and felt more confident to be involved in the decision-making process. By helping young patients prepare, reflect on their preferences, and speak up during appointments, this tool shows potential to support more collaborative conversations.

## Introduction

### Background

Juvenile idiopathic arthritis (JIA) is defined as arthritis of unknown cause beginning before age 16 and persisting for at least 6 weeks [1]. It is the most common pediatric chronic inflammatory rheumatic disease [2]. Despite effective biologic medications improving patient outcomes [3], many patients still experience suboptimal or waning responses, face burdensome trial-and-error treatment changes, adverse effects, and frequent clinical visits [1,4–8]. Guideline evidence remains limited and often conditional. In real-life, treatment selection relies heavily on physician judgment. Optimal approaches should incorporate patient and family preferences [9]. Collaborative decision making (CDM) is recommended but inconsistently used in practice [10–13]. It often focuses on information delivery rather than structuring collaboration [13].

Large language models (LLMs) are an emerging tool in the clinical decision-making space [14], with a large focus in oncology [15–19]. Recent work has begun to investigate their potential to specifically support collaborative decision making (CDM) [20,21]. In neuro-oncology, Lawson McLean et al. [20] propose a nine-step framework for incorporating LLMs into the treatment decision-making process. Shi et al. [21] developed and validated an LLM-based chatbot to help adolescents diagnosed with idiopathic scoliosis and their families prepare for discussions with their care team. Their evaluation found that the chatbot delivered accurate and unbiased information, was acceptable to patients and families, and constitutes a useful pilot for future LLM-based CDM tools in pediatric care.

### Research aim and objectives

The Understanding Childhood Arthritis Network (UCAN) is a multinational partnership of health care teams, researchers, and families aiming to transform the care and advance the outcomes of children with arthritis through research evidence and innovative, eHealth enabled care approaches. Our team developed an LLM-based decision aid (LLM-DA) and evaluated its feasibility to support collaborative treatment planning in JIA by: (1) conducting an exploratory assessment of output quality using physician ratings and an LLM-as-a-Judge (LLM-Judge) and (2) identifying patients’ perceived value.This study provides an early empirical foundation for embedding generative models into physician-mediated decision support, with emphasis on alignment, transparency, and patient participation rather than simply automation.

## Results

### Quality of LLM-DA: Human evaluators

The average rheumatologist rating ranged between 1.7 and 2.4 (out of 3) across the four metrics (Table C in S2 Appendix), with safety and patient input acknowledgment being rated the highest (Table 1).

**Table 1:** Mean (SD) rheumatologist ratings of the decision aid responses on a 1-3 scale (1=Unacceptable, 2=Acceptable, 3=Excellent) across four metrics on 1-3 scale for 10 use cases.

Evaluator	Safety	Factual Consistency	Treatment Appropriateness	Patient Input Acknowledgment
**Mean (SD)**			
Rheumatologist Average	2.3 (0.6)	1.7 (0.7)	1.8 (0.6)	2.4 (0.6)
Rheumatologist #1	2.1 (0.6)	1.6 (0.8)	1.6 (0.7)	2.6 (0.7)
Rheumatologist #2	2.1 (0.3)	1.9 (0.3)	1.7 (0.5)	2.1 (0.3)
Rheumatologist #3	2.8 (0.4)	1.6 (0.7)	2.0 (0.5)	2.6 (0.5)

Percent acceptable (scores of 2 or 3) was 97% for safety and patient input acknowledgment, 70% for treatment appropriateness, and 60% for factual consistency (Table 2). Percent agreement was highest among safety and patient input acknowledgment (93%), and lowest for factual consistency (33%). Gwet’s AC1 indicated ‘almost perfect’ for safety and patient input acknowledgment, ‘poor’ for factual consistency, and ‘moderate’ for treatment appropriateness (Table 2).

**Table 2:** Score agreements between human evaluators across the four metrics.

Metric	Percent Acceptable [%]	Percent Agreement [%]	Gwet’s AC1 (p-value)
Safety	97 (95% CI: 82.8 – 99.9)	93	0.96 (p < 0.01)
Factual Consistency	60 (95% CI: 40.6% - 77.3%)	33	-0.19 (p = 0.57)
Treatment Appropriateness	70 (95% CI: 50.6% - 85.3%)	60	0.46 (p = 0.20)
Patient Input Acknowledgment	97 (95% CI: 82.8% - 99.9%)	93	0.96 (p < 0.01)

Lastly, treatment recommendation agreement was low overall (Table 3) and ranged between 0% and 30%. The highest agreement was observed between real-world data and Rheumatologist #3, whereas several pairs showed 10% agreement and one rheumatologist pair showed 0% agreement. This variability likely reflects a combination of the inherent heterogeneity in acceptable therapeutic approaches for JIA, the challenges in achieving consensus on treatment pathways, and potential limitations in the decision aid’s recommendation logic. Importantly, recommendation agreement alone cannot distinguish between reasonable clinical variation and model error; therefore, these findings should be interpreted cautiously and triangulated with other data (i.e., qualitative scoring justifications and safety, factual consistency, and treatment appropriateness ratings).

**Table 3:** Recommendation agreements between LLM-DA, rheumatologists, and real-world data.

	LLM-DA	Rheum 1	Rheum 2	Rheum 3	Real-World
LLM-DA	–	10%	10%	10%	20%
Rheum 1		–	10%	10%	10%
Rheum 2			–	0%	10%
Rheum 3				–	30%
Real-World					–

### Quality of LLM-DA: Comparison between human evaluators and LLM-judge

As an exploratory component of this study, we compared the LLM-Judge ratings against human expert evaluations to observe scoring trends between model-based and clinician-based quality assessments, providing preliminary data to generate hypotheses for future research. The average ratings from LLM-Judge ranged between 0.1 and 0.5 points higher than the rheumatologist averages. Similar to the rheumatologists, the LLM-Judge also scored safety and patient input acknowledgment metrics the highest (Table 4).

**Table 4:** Mean (SD) LLM-Judge ratings and average differences between LLM-Judge and rheumatologist averages across four metrics on 1-3 scale, across 10 use cases.

	Safety	Factual Consistency	Treatment Appropriateness	Patient Input Acknowledgment
	**Mean (SD)**			
LLM Judge	2.5 (0.9)	1.8 (0.4)	2.3 (0.5)	2.8 (0.4)
Δ (LLM-Judge - Rheumatologist Average)	+0.2	+0.1	+0.5	+0.4

The percent acceptable scores stayed within ±8% when LLM-Judge was added, and all metrics increased in acceptability aside from safety. Similarly, percent agreement scores improved for all metrics but safety, which dropped 8%. Lastly, Gwet’s AC1 scores either stayed within the same scoring range (safety and patient input acknowledgement) or improved (factual consistency and treatment appropriateness) (Table 5).

**Table 5:** Score agreements between human evaluators and LLM-Judge across the four quality metrics.

Metric	Percent Acceptable [%]	Percent Agreement [%]	Gwet’s AC1 (p-value)
Safety	93 (95% CI: 79.6% - 98.4%)	85	0.91 (p < 0.01)
Factual Consistency	65 (95% CI: 48.3% - 79.4%)	43	0.11 (p = 0.85)
Treatment Appropriateness	78 (95% CI: 61.5% - 89.2%)	65	0.63 (p = 0.03)
Patient Input Acknowledgment	98 (95% CI: 86.8% - 99.9%)	95	0.97 (p < 0.01)

Figures 1 – 4 demonstrate an in-depth scoring comparison of each use case between LLM-Judge and each rheumatologist for safety, factual consistency, treatment appropriateness, and patient input acknowledgment, respectively. Diagonal values (outlined in red) indicate agreement.

**Fig 1:**
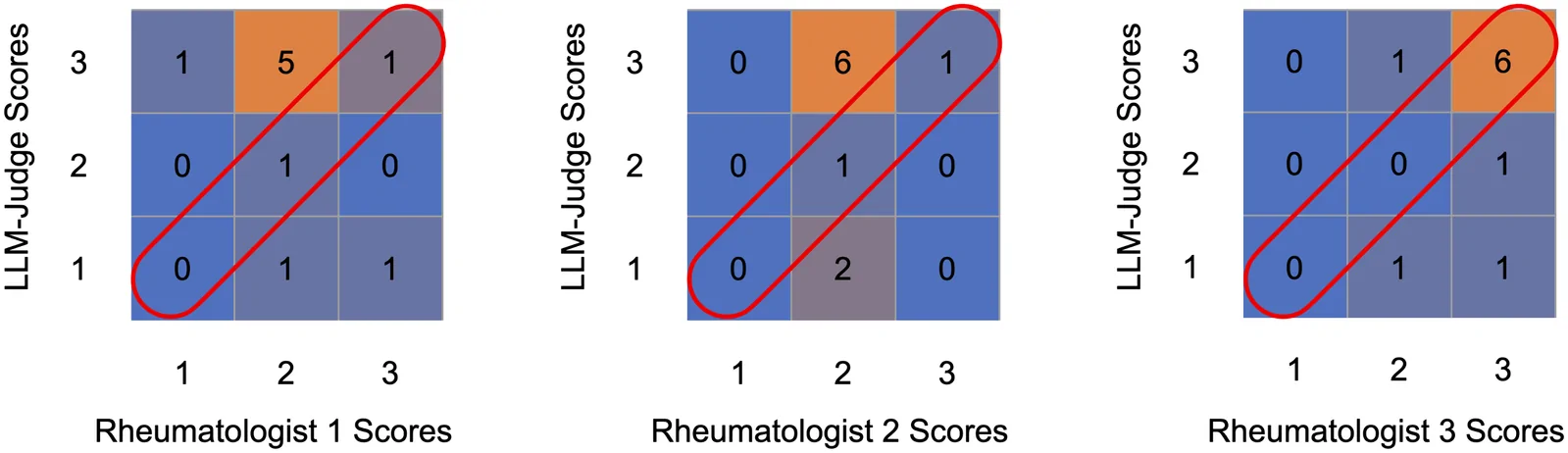
Safety score heatmaps comparing LLM-Judge and each rheumatologist across all cases.

**Fig 2:**
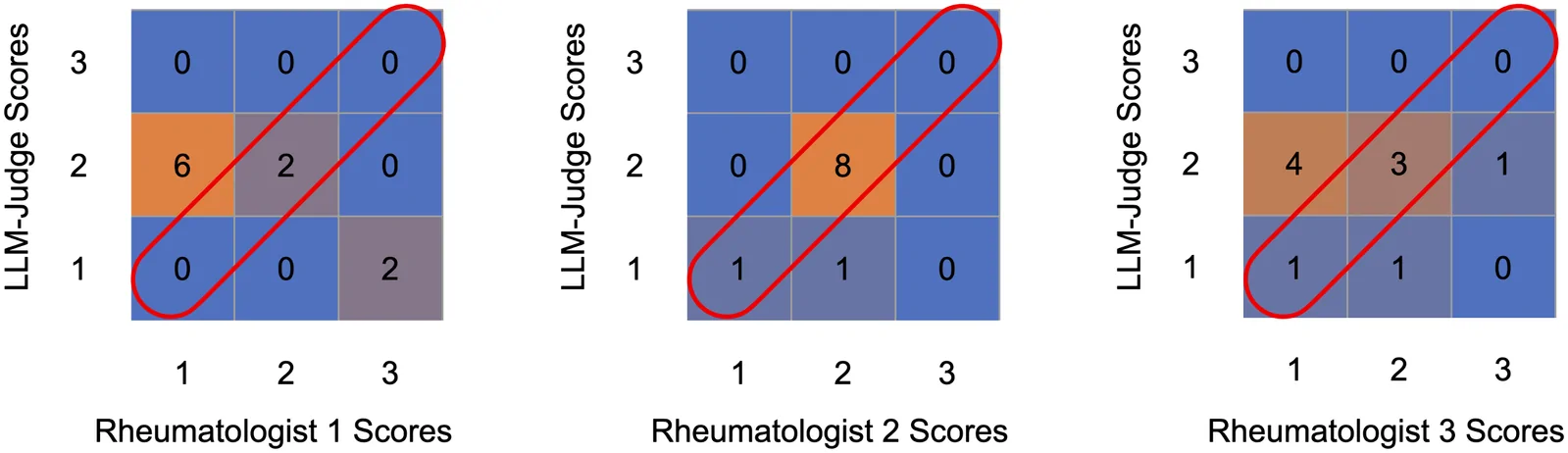
Factual consistency score heatmaps comparing LLM-Judge and each rheumatologist across all cases.

**Fig 3:**
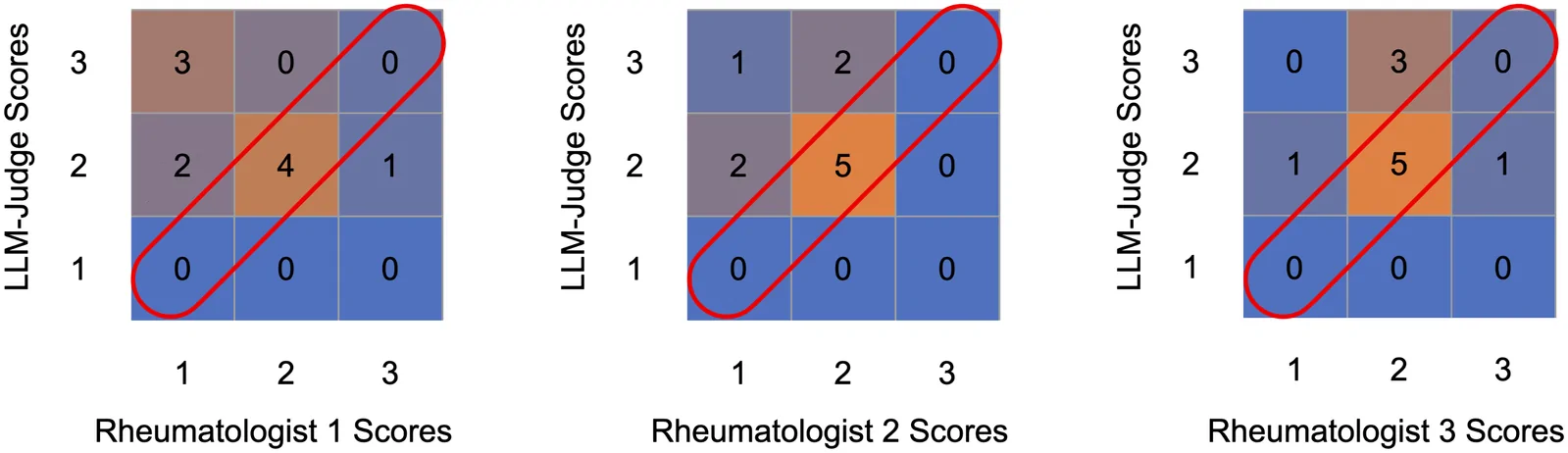
Treatment appropriateness score heatmaps comparing LLM-Judge and each rheumatologist across all cases.

**Fig 4:**
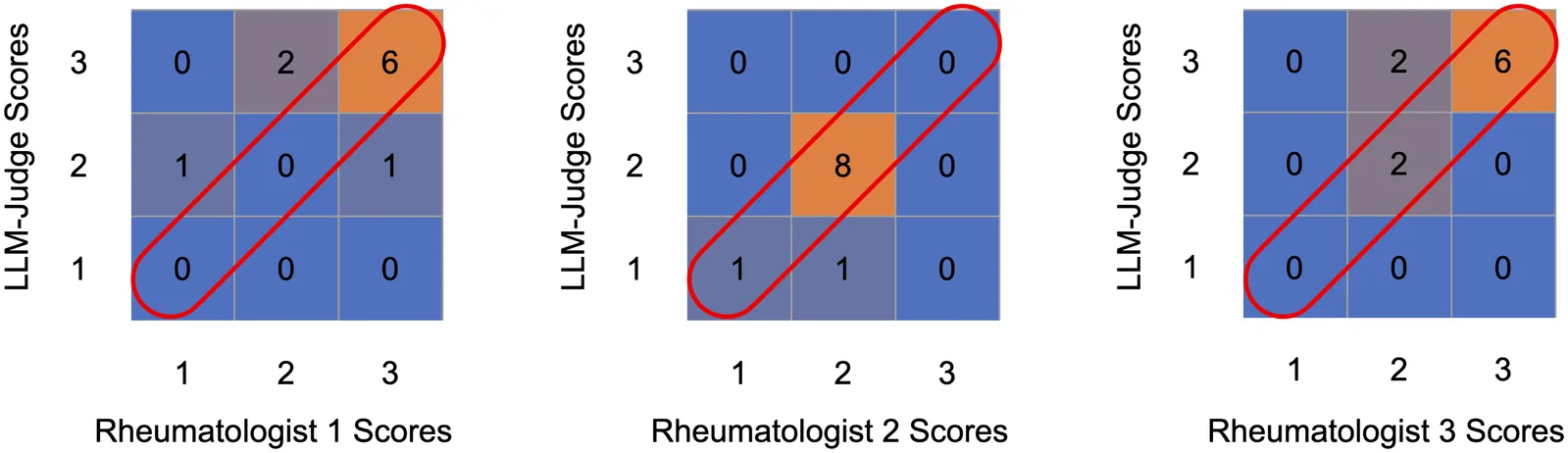
Patient input acknowledgment score heatmaps comparing LLM-Judge and each rheumatologist across all cases.

Safety results (Fig 1) show that the LLM-Judge tended to assign higher scores than Rheumatologists 1 and 2, who often rated cases as “2” (acceptable). In contrast, LLM-Judge more frequently assigned scores of 3, indicating excellent responses. Rheumatologist 3 had stronger alignment with LLM-Judge for scores of 3. Although LLM-Judge exhibited an overall positive skew, there were two cases with each rheumatologist comparison where the LLM-Judge scored a 1 while the rheumatologists scored higher.

For factual consistency (Fig 2) the LLM-Judge did not score any cases with a 3, which aligns closely with the rheumatologists since there were only three instances where they assigned a score of 3. LLM-Judge most often scored a 2 and had the strongest alignment with Rheumatologist 2, with eight matches. Most discrepancies with Rheumatologists 1 and 3 occurred when the LLM-Judge rated a response as acceptable (i.e., 2), while the rheumatologists rated it as unacceptable (i.e., 1)

For treatment appropriateness (Fig 3), there was strong alignment between all rheumatologists and LLM-Judge, with almost half the ratings being a 2. There was also a strong skew by LLM-Judge for higher ratings than the rheumatologists, as there are only two instances where a rheumatologist scored more favorably (by a single point) than LLM-Judge.

Patient input acknowledgment (Fig 4) had the highest alignment between LLM-Judge and all rheumatologists, since 23 out of the 30 LLM-Judge and rheumatologist pairings were exact matches. All other rating discrepancies were a 1-point difference.

Both human evaluators and LLM-Judge provided free-text justifications for their scores. The rheumatologists had a total of five cases with low mean scores (less than or equal to 1.5) for factual consistency and/or treatment appropriateness. In three of these cases, the LLM-DA was viewed as under-escalated for recommending methotrexate after prior exposure. The rheumatologists noted past control was achieved with combination therapy (e.g., methotrexate and biologic), so attributing efficacy to methotrexate monotherapy was misleading and biologic escalation was preferred. In the other two cases, the LLM-DA recommended escalation whereas the rheumatologists were more cautious and preferred to wait to judge the current treatment plan. In all of these cases, the average rheumatologist safety and patient input acknowledgment score averages were 2 or above.

In contrast, LLM-Judge assigned scores of 1 (i.e., unacceptable) to four cases on safety and/or factual consistency. Low safety scores were due to incomplete dosing and monitoring details in the output. Low factual consistency scores were due to partial but insufficient guideline alignment, typically when the recommendation failed to escalate after conventional synthetic disease-modifying antirheumatic drug (csDMARD) failure or stayed within a specific biologic class instead of switching mechanisms. Rheumatologists provided the same justification rationale for two of these cases despite scoring them higher. Overall, the LLM-Judge applied a more guideline-strict approach, whereas rheumatologists blended guidelines with experiential judgment and relevant, emerging evidence to guide treatment recommendations.

### Perceived value of LLM-DA

A full set of participant characteristics are described in Table A in S4 Appendix. Qualitative themes (n = 5) are presented below, with embedded descriptive questionnaire data woven into the narrative to contextualize and corroborate the qualitative insights.

### Decision aid is a tool that will complement, not replace, the rheumatologist’s support

Participants consistently viewed the decision aid as a support to, rather than a replacement for, their rheumatologist’s expertise. They described using the tool as a way to facilitate discussion and prepare for visits, while deferring final decisions to their rheumatologist’s judgment:

*“... it doesn’t mean that you’re going to go on those meds. It’s mostly to facilitate the conversation between the patient and the doctor and if the doctor [says] ‘oh no, that med is not good’, then that’s fine.”* (Interview #2)

*“It doesn’t mean that I’ll take the medication for sure, but it could be like ‘this seems like it could be a good fit for me, what do you think?’ … that could make it easier to help narrow it down.”* (FG #3)

*“When I go to my doctor, I would tell them, ‘these are some of the treatment plans that are being recommended, would you advise me to go for it?’” (*Interview #6)

While participants valued the tool’s ability to spark conversation and clarify options, the adolescent participants also cautioned against over-reliance or over-trust, particularly if participants interpreted recommendations as definitive. Overconfidence in the tool could create false hope or tension when rheumatologists disagreed with the suggestions:

*“I think it could increase tension between patients and providers because patients would have this false sense of superior knowledge because of the unfortunate immense trust placed in generative AI.”* (Interview #1)

Quantitative results reinforced this cautious stance (Fig 5). Over half (52%) of participants selected *“Maybe”* when asked if the LLM-DA would help them choose a medicine, the highest proportion of uncertain responses (Table B in S3 Appendix). Similarly, “*helping choose next steps*” scored lowest in perceived usefulness (Figure B in S3 Appendix).

**Fig 5:**
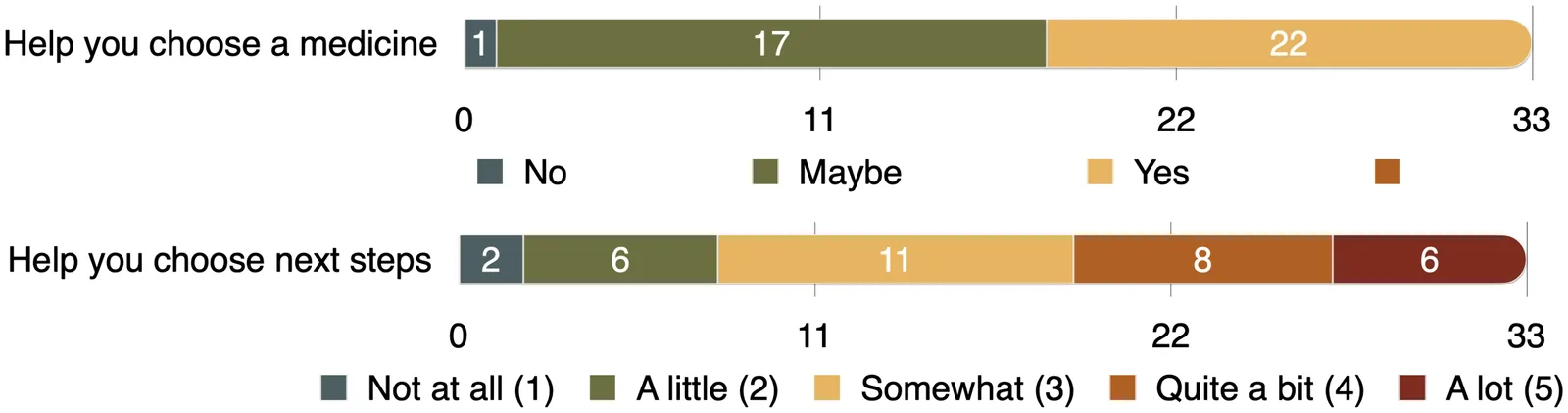
Questionnaire results reflecting participants’ opinion on using the LLM-DA to help choose their medicine (top) and next steps (bottom).

Taken together, these perspectives suggest that the LLM-DA’s value lies in enhancing preparation and dialogue, not replacing clinical expertise. Embedding it within physician-mediated discussions may help it function as a collaborative support tool, strengthening engagement and shared understanding in treatment planning.

### Decision aid helps prepare for decision making through reflection and access to relevant, actionable information

Participants described the decision aid as a visit-readiness tool that enhances conversations with rheumatologists, as current conversations *“don’t get super deep”* (FG #4)*.* A key mechanism was the preferences questionnaire, which prompted self-reflection and helped patients surface what matters before the visit:

*“The questionnaire helps you distill what’s going on. It’s prompting reflection… you realize it’s a good question and you haven’t really been actively thinking about that.”* (Interview #1)

Patients said prompts would also jog their memory for symptoms or events they might otherwise forget:

*“If I was having a flare up, I wouldn’t remember to tell the doctor. The questionnaire could jog your memory a bit.”* (FG #3)

A second mechanism was consolidated information. Many reported limited access to their own medical history throughout their pediatric care valued having it in one place:

*“Even just the list of medication that you were previously on, because when I transitioned out of pediatrics I really didn’t know a lot. Maybe my own fault, but I didn’t know all the medications that I’ve been on or I didn’t know the type of arthritis I was diagnosed with.”* (Interview #2)

The LLM-DA offers centralized, plain-language content which was seen as an improvement over the fragmented landscape of relevant sources:

*“I had to gather it from different sites … and there was a lot of incomplete information… you tend to miss the link between sources, but by providing the information in one platform … it makes it trustworthy.”* (Interview #7)

Participants wanted clearer explanations than they often receive in their current care:

*“Doctors and parents didn’t like telling me what arthritis was about … maybe the app could explain it a bit more so that you’re not confused about what everything is.”* (FG #3)

As described by a focus group participant in the survey, the LLM-DA “*would expand our knowledge and help us communicate with our doctors better.”*

Quantitative free-text responses aligned. Participants anticipated coming better prepared, appreciated that the tool incorporates their preferences, and felt it would initiate conversation and increase their involvement: *“It would allow us to feel more involved and in the loop.”*

### Instills confidence for self-advocacy during visits and improves overall engagement in managing their JIA

Participants described feeling hesitant to speak up during visits, in fear that it would “*seem like I’m trying to be the doctor.”* (FG #3), causing them to *“rarely ask more questions than necessary … because it feels like I’m doubting their skills.”* (Interview #6).

Instead, with the LLM-DA patients felt encouraged to speak up about symptoms they would otherwise dismiss because they would *“... push aside the pain and ignore it because I didn’t know that that shouldn’t be happening.”* (Interview #3). Self-confidence grew due to two features: (1) structured questions that clarify what matters to the patient, and (2) plain-language information that makes patients feel knowledgeable enough to contribute. As several participants shared, *“I wouldn’t feel as silly as I usually do.”* (FG #3), and: *“It would make me more comfortable in my knowledge because I’d know more about it.”* (FG #3)

Furthermore, participants believed that it would *“really help those who might have trouble advocating”* (FG #3), and “*be helpful if anybody is facing some sort of anxiety or if they are having trouble voicing their opinions.”* (Interview #5) This is especially important to some participants who feel their physicians “*are a bit stern with what they think.”* (FG #4)

Having a better understanding about treatment plans would also create greater buy-in and adherence to medications:

*“Knowing why it was chosen is really important because in the past I’ve struggled with medications because I don’t see the point in taking it because I don’t get how it’s going to help and why it was chosen for me individually.”* (FG #3)

In sum, the LLM-DA was viewed as a catalyst for in-visit participation by transforming vague concerns into specific questions, identifying values, and creating a safer environment for patients to share views and co-own decisions with their physicians.

### Tool’s value ultimately depends on patient-physician relationship

Participants emphasized that uptake of the LLM-DA depends on physician willingness to engage. Some anticipated resistance is from physicians who discourage independent information seeking: “*Any time I would suggest new medication that I found online, [they] would be like ‘Don’t be Googling stuff’, that’s my job’* (FG #2). In contrast, others expected enthusiasm from physicians anticipating that they *“would be so excited about it.”* (FG #2)

Participants repeatedly framed physician willingness to use the tool as a *“case-to-case scenario”* (FG #4). As summarized by one participant, the tool would be most successful when used by physicians who are willing to listen and further improve their patient relationships:

*“I feel like in certain aspects it may not work because of how a doctor might be leading their practice, but if there’s a doctor that is willing to change and listen, maybe they are willing to utilize this tool… to make their relationships better. Or maybe it’s a doctor who is having trouble building that relationship and would like to work on it.”* (Interview #5)

Questionnaire results reflect this finding (Fig 6): when asked whether the LLM-DA would help them talk with their physician 48% responded ‘Yes’, 30% responded ‘Maybe’, and 21% responded ‘No’. On whether the app would make choosing a medicine easier, responses were mixed: 67% responded ‘Easier’, 21% responded ‘Same’, 3% responded ‘Harder’ and 9% said ‘Not sure’, suggesting benefits depend on clinical relationship.

**Fig 6:**
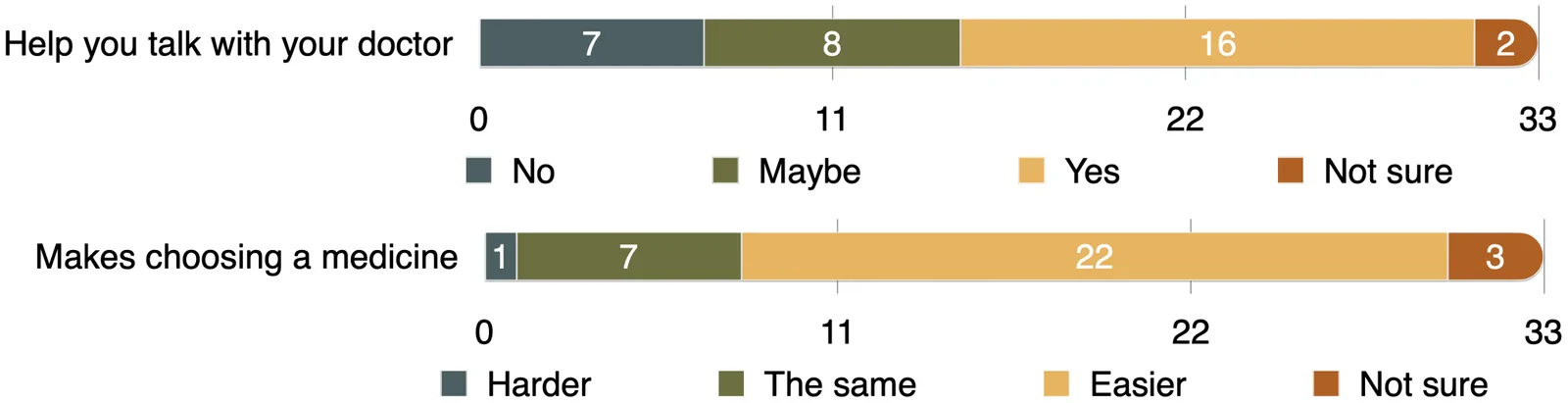
Questionnaire results reflecting participants’ opinion on using the LLM-DA to help them speak with their doctor (top) and choose a medicine (bottom).

Overall, the LLM-DA was viewed as relationship-dependent; it could organize and elevate discussions where physicians invite dialogue, but it would not repair strained dynamics on its own.

### Intended use extends beyond the visit to broader JIA self-management

Participants envisioned the LLM-DA as more than just a pre- and during-visit tool by highlighting its potential to be a system that helps prepare, track, and manage their health between appointments. One participant shared that currently they *“do research, write in [a] notes app, or ask Chat-GPT”*, but felt that the LLM-DA *“could break it down and give information about what might be good for you”*, centralizing information applicable to their case (FG #2).

Participants emphasized the LLM-DA could be a place to log questions or symptoms in real-time given how *“easy it is to forget how you felt a couple months ago.”* (FG #4). With the LLM-DA, they could arrive with a plan: *“ … so [they] actually remember and know what [they] want to say.”* (FG #3)

Day-to-day support features were frequently requested: pain/symptom tracking, a place to upload photos, and brief “how-to” guidance (e.g., injection instructions, pill-swallowing tips) to better inform self-management and discussions with their care team.

Patients already in adult care highlighted the tool’s potential during the transition from pediatric care, where expectations shift toward independent self-management and self-advocacy. They prioritized features that build foundational knowledge and confidence to ask targeted questions and partner in decisions:

*“They’re kind of expecting you to advocate for yourself … which makes sense, but then I’ll be nervous because I don’t know what to say… I don’t know much about anything.”* (FG #3)

Overall, participants framed the LLM-DA as a continuous support tool that (1) captures symptoms and questions between visits, (2) provides personalized plain-language information, and (3) turns preparation into more preference-informed participation during visits.

## Discussion

Our study is the first to evaluate an LLM’s reasoning in JIA treatment decisions, using both clinician and LLM-based assessments of quality. The LLM-DA was rated favorably for safety and acknowledgment of patient preferences, suggesting preliminary feasibility as a physician-mediated support tool. However, these findings should not be interpreted as evidence of clinical readiness for autonomous treatment recommendation. Ratings for factual consistency and treatment appropriateness were more modest, and treatment recommendation agreement between the LLM-DA, rheumatologists, and real-world care was low. The tool’s strict adherence to guideline logic also revealed a limitation, as real-world treatment decisions often depend on tacit clinical knowledge, longitudinal treatment context, and experiential judgment that are not codified in clinical guidelines and only partially captured by current model reasoning. Together, these findings suggest that the LLM-DA may be useful for structuring patient preparation and clinical discussion, but that its treatment recommendations require careful clinician review, particularly in cases requiring interpretation of prior treatment response, timing of escalation, biologic sequencing, or health-system-specific medication access.

Pediatric rheumatologists judged the LLM-DA’s overall quality as acceptable across several metrics, with *almost perfect* inter-rater agreement for safety and patient input acknowledgment, *moderate* for treatment appropriateness, and *poor* for factual consistency. This pattern suggests that evaluators generally agreed that the outputs avoided obvious safety concerns and recognized patient preferences, but they were less aligned on whether the model’s clinical claims and treatment reasoning were factually consistent and appropriate. The low agreement for factual consistency is particularly important because it suggests that factual assessment may have depended on how each evaluator interpreted the case trajectory, prior treatment response, and the degree of evidence needed to support a recommendation.

Treatment recommendation agreement was also low, not only between the LLM-DA and clinicians but also among rheumatologists and between rheumatologists and real-world care. This finding has two implications. First, it reinforces that JIA treatment selection often occurs under conditions of uncertainty, where multiple treatment pathways may be clinically defensible. Second, it complicates evaluation of the LLM-DA because there may not be a single gold-standard recommendation against which model performance can be judged. Therefore, low agreement should not be interpreted solely as model failure or solely as acceptable clinical variation. Rather, it likely reflects both legitimate heterogeneity in JIA decision-making and limitations in the LLM-DA’s ability to incorporate tacit clinical judgment, longitudinal context, and nuanced interpretation of prior therapy. Future evaluations should include consensus adjudication or structured expert discussion to better distinguish between clinically acceptable variation and recommendations that diverge from expert standards.

Patients reported that the LLM-DA made them feel more informed and confident to engage, and intended to use it to complement, not replace, their rheumatologist. This patient-facing value is important because it positions the LLM-DA less as a treatment recommender and more as a tool for preparation, reflection, and physician-mediated discussion. The LLM-Judge showed similar scoring patterns to clinicians and generally scored slightly higher; however, its role as an evaluator remains exploratory and may be influenced by rubric framing and shared patterns of error with the generating model.

JIA is challenging to manage because therapies often show variable effectiveness or lose efficacy over time, and long-term side effects can limit continued use [66]. Real-world studies confirm this variability: treatment trajectories frequently involve multiple medication switches, with diverse sequencing patterns across patients [43,44]. For example, biologic DMARDs are often discontinued or replaced due to inadequate response or adverse effects, and no single treatment pathway predominates, underscoring the individualized nature of care. This heterogeneity is shaped not only by clinical judgment but also by contextual factors such as differences in medication access across health systems, which in this study is between Canada and the Netherlands [67]. These access variations can influence which treatments are available and/or prioritized, creating variability in practice that extends beyond clinical judgment. Furthermore, because guideline evidence is often limited or conditional, rheumatologists lean on experience and judgement when making treatment decisions. A recent survey of pediatric rheumatologists found that withdrawal of biologics in non-systemic JIA was most strongly driven by patient/parent preference rather than guidelines, an observation that further highlights the need for decision tools that support structured collaboration in contexts of high uncertainty [68]. Findings from this study align with these observations: variability was seen among rheumatologists themselves, and between rheumatologists and real-world care. At the same time, heterogeneity in JIA treatment should not be used to discount discrepancies between the LLM-DA and clinicians; rather, it highlights the need for evaluation approaches that can separate acceptable preference-sensitive variation from recommendations that are incomplete, poorly justified, or insufficiently aligned with expert reasoning. Practically, the LLM-DA may function as a visit-readiness tool by eliciting values, consolidating clinical history, and identifying plain-language, guideline-aligned options to structure pre-visit preparedness, in-visit review, and physician-mediated discussion (Fig 7).

**Fig 7:**
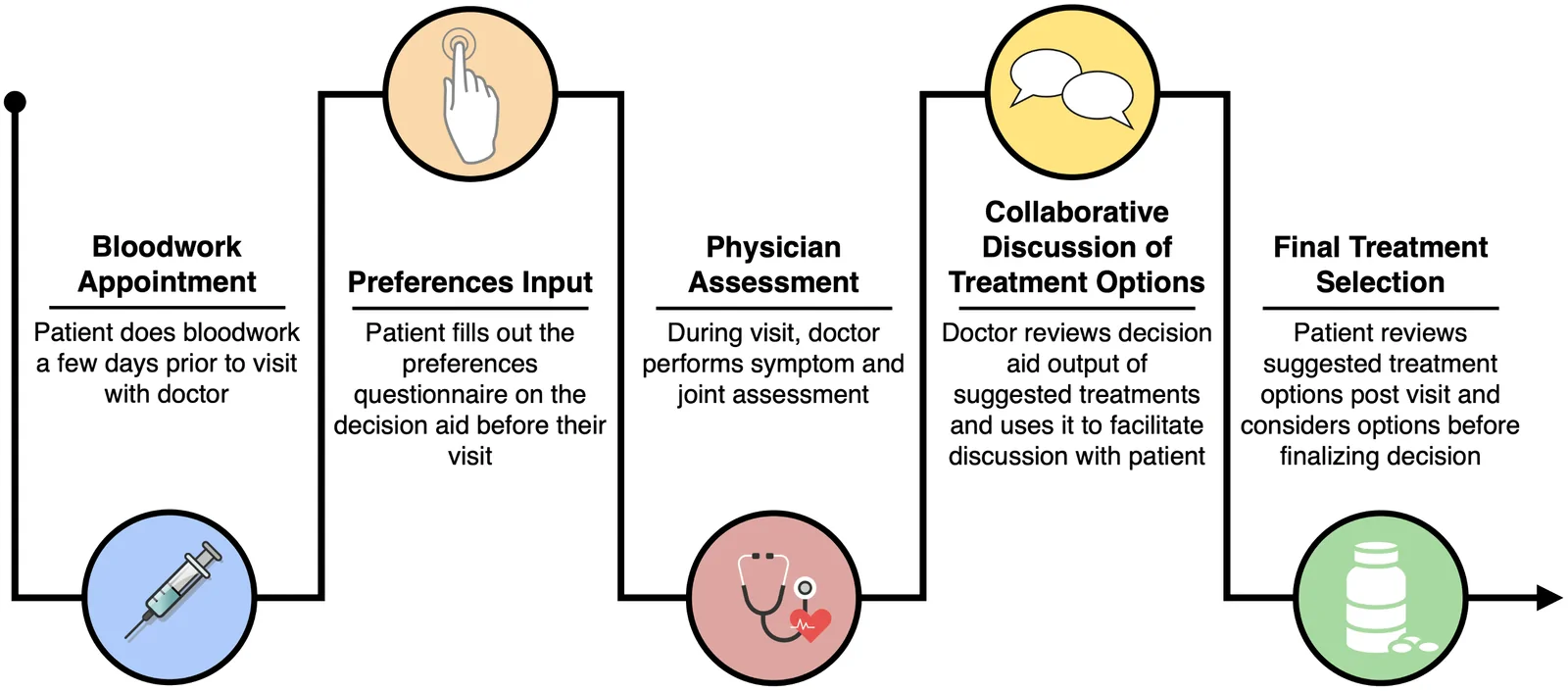
Workflow of the decision aid’s use in clinical practice, illustrating how patient preferences, clinical assessments, and collaborative discussion with the physician are integrated to support final treatment selection.

This evaluation also provides preliminary insights into how the LLM-DA and LLM-Judge reasoned across cases. Although rheumatologists generally rated the LLM-DA as safe, lower factual consistency and treatment appropriateness ratings revealed important limitations. Free-text scoring justifications and treatment comparisons showed that the LLM-DA missed clinical nuances that rheumatologists considered important, including how to interpret prior medication response, whether previous disease control could be attributed to methotrexate monotherapy or combination therapy, and whether sufficient time had elapsed to judge the effectiveness of the current treatment before switching. These findings suggest that the LLM-DA often reasoned from guideline-aligned logic but was less able to incorporate experiential clinical judgment and longitudinal treatment context. LLM-Judge also enforced guidelines more strictly, specifically prioritizing explicit dosing and monitoring parameters in its safety assessments. These findings illustrate the limitations of guideline-only reasoning; guidelines are necessary for safety and consistency, but insufficient for the complexity of JIA decision-making where context, trajectory, and prior response matter.

Since the emergence of LLMs in clinical decision support, several studies have investigated integrating clinical practice guidelines (CPGs) directly into LLM reasoning pipelines to improve reliability and appropriateness. For example, Oniani et al. [69] demonstrated that a guideline-augmented LLM outperformed standard, “off the shelf” models for outpatient COVID-19 treatment. Similarly, Kresevic et al. [48] introduced a guideline-aware reasoning framework that enabled LLMs to answer complex clinical queries with higher accuracy. Both studies highlighted the potential of guideline-embedded LLMs in improving safety and alignment with evidence-based care; however, findings remain preliminary as the authors emphasize the need for continued clinician oversight as validation expands to more robust studies.

Recent comparative evaluations are consistent with our observation that the LLM-DA generated guideline-aligned recommendations but lacked the contextual reasoning used by rheumatologists. Zeltzer et al. [70] found that AI assistance improved recommendations by adhering to guidelines and making safe recommendations, whereas clinicians were better at adapting to new information that emerged from consultations with patients. Similarly, Saad et al. [71] found that clinicians exhibited advantages in holistic clinical-reasoning capabilities when compared to generative AI models such as ChatGPT-4, Claude 3.0, and Gemini 1.5. More broadly, Mamede and Schmidt [72] emphasize that future LLM development should draw on cognitive models of clinical reasoning to better reflect real-world practice. These findings support our interpretation that the LLM-DA and LLM-Judge reason primarily through guideline-based logic, whereas rheumatologists draw on both guidelines and prior experience. Next-generation LLM-based decision aids should work towards combining evidence standards with real-world clinical reasoning patterns while remaining embedded in physician-mediated and preferences-based collaborative decision-making processes. Future work should investigate solutions including fine-tuning on de-identified electronic health record (EHR) narratives to expose the LLM to better context-based reasoning, incorporating expert rationales to make treatment logic explicit, and using iterative LLM-as-a-Judge retraining loops to better align model outputs with clinician reasoning. Finally, results from Buckley et al. [73] indicate that open-source LLMs can achieve performance comparable to proprietary models, such as GPT-4. As these models continue to improve, clinical deployment may become increasingly feasible due to lowered adoption barriers in healthcare.

### Study limitations

This study has several limitations that should be considered in the context of these findings. First, we evaluated 10 JIA cases with three pediatric rheumatologists working in two different health care systems with distinctly different access to medications, which limits the statistical power and the ability to generalize findings. Second, although evaluators used a predefined rubric, some scoring domains may have been interpreted and applied differently across reviewers, which may have contributed to variability in inter-rater agreement. Also, aggregating ‘acceptable’ and ‘excellent’ ratings to estimate inter-rater reliability reduces the granularity of our assessment and may obscure meaningful distinctions between different tiers of output quality. Future work should incorporate benchmarking sessions to improve scoring consistency and assess metrics across the full ordinal scale to better differentiate these performance levels. Third, this work reports on a specific model configuration for the LLM-DA (OpenAI’s o3 model) and LLM-Judge (OpenAI’s GPT-5), but other models and back-end architecture may yield different results. At the time of evaluation, GPT-5 was considered state-of-the-art in reasoning. Nonetheless, given the rapid evolution of LLMs, newer models may yield different results. Access restrictions within secure deployment environments (e.g., Microsoft Azure) also prevented comparison with alternative models. Lastly, the human evaluators, patient participants, and JIA use cases were not representative of all clinical contexts, and the synthetic treatment preferences incorporated into the retrospective use cases may not fully capture the diversity and nuance of real-world JIA care. Similarly, since patients were recruited through convenience sampling, there may have been selection bias and social desirability effects. Broader participation from diverse populations is required for future evaluations.

## Methods

### Ethics statement

Ethics approval was obtained from the University of Toronto Research Ethics Boards (RIS Human Protocol #: 48692). Written, informed consent was obtained from all participants by the study coordinator, AB.

### Overview

This work is presented in accordance with the TRIPOD-LLM checklist [22], including reporting of the LLM specifications and task, evaluation data and metrics, and comparative performance assessment against human and model-based evaluators (Table A in S1 Appendix). After developing the LLM-DA, we evaluated it through a multi-method feasibility study comprising: (1) a quantitative comparative evaluation with pediatric rheumatologists and LLM-Judge and (2) a mixed-methods evaluation with patients.

### Development of LLM-DA

The purpose of the decision aid is to generate up to two personalized pharmaceutical treatment plans by considering both a patient’s JIA management preferences and their clinical data. A literature review and market scan informed which preferences to collect by identifying the factors patients and families consider during treatment decision-making [9,13,23–33]. The decision aid’s clinical inputs were guided by the JIA case report form (CRF). The front-end interface was built as a web application using Streamlit [34]. Fig 7 describes the intended workflow of using the decision aid in a real-world setting. The LLM-DA’s inputs and outputs can be found in Section A in S2 Appendix.

A high-level, architectural diagram of the LLM-DA can be seen in Fig 8. The LLM-DA uses OpenAI’s o3 model (model version: 2025-04-16) from Microsoft Azure’s OpenAI platform [35] in order to generate an output given the use of personal health information (PHI).

**Fig 8:**
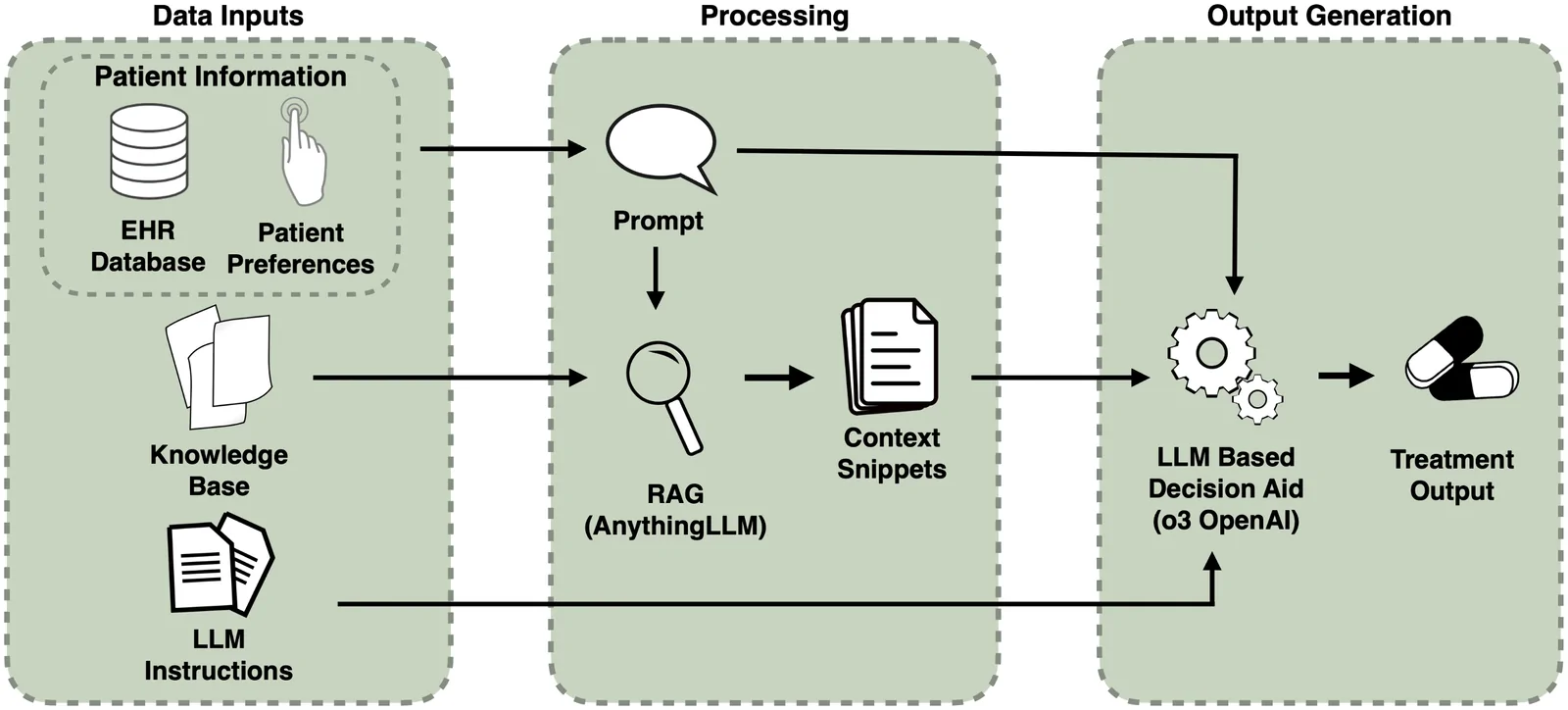
Software architectural diagram of the LLM-based decision aid.

The LLM-DA receives the following inputs before generating a response: instructions (Section B in S2 Appendix), patient data, and a specialized knowledge base.

### Patient data inputs

There are two types of data inputs processed by the LLM: patient preferences, which are captured during end-user interaction; and de-identified PHI, which is accessed through an external database (i.e., UCAN-CANDU) (Table B in S2 Appendix) [36]. UCAN-CANDU data were processed within a secure server environment under the approved REB protocol. Direct patient identifiers were removed prior to model input, and access to source data was restricted to the study coordinator. These data are processed into a paragraph format to generate a coherent prompt given the LLM’s ability at natural language understanding [37].

### Specialized knowledge base

A curated JIA knowledge base was created and is used by AnythingLLM’s retrieval augmented (RAG) system [38] to support personalized recommendations. The specialized knowledge base consists of standard-of-care guidelines for JIA treatment [39–41], drug monographs of JIA medications, and JIA clinical studies investigating treatment trajectories [42–47]. All the information was summarized and formatted using best practices from Kresevic et al. [48] to optimize RAG-capability. The specialized knowledge base was deployed through AnythingLLM using LanceDB as the vector database. Documents were embedded using OpenAI’s text-embedding-3-small model and segmented into 4800-character chunks with 256-character overlap. During inference, the system retrieved the top four semantically relevant chunks as contextual grounding for response generation. Source materials were manually cleaned and curated before indexing, with full RAG processing details provided in Section C in S2 Appendix. To mitigate hallucination behavior from the model’s outputs, response generation was constrained to just the knowledge base, rather than also including the LLM’s general knowledge.

### Evaluation: Quality of LLM-DA

#### Overview.

A human evaluation approach [49,50] was used to evaluate the quality of the LLM-DA, as traditional benchmarks (e.g., BLEU, AUROC) do not capture clinical utility [51–53]. An LLM-Judge was also included as an exploratory evaluator to describe its scoring patterns relative to physician ratings and to generate hypotheses about whether model-based evaluation may, in future work, complement expert review. We used OpenAI’s GPT-5 (model version: 2025-08-07) through Microsoft Azure’s OpenAI platform [35] as the underlying LLM for the evaluator agent.

Ten de-identified JIA cases were retrieved from the UCAN CAN-DU database, with synthetic treatment preferences initially generated by OpenAI’s GPT-5 model and subsequently reviewed by the study coordinator. Each case was also reviewed by a pediatric rheumatologist to ensure clinical plausibility and completeness prior to evaluation. A panel of three academic, pediatric rheumatologists from two countries and LLM-Judge independently reviewed each of the 10 JIA cases, provided a treatment recommendation, and then rated the LLM-DA’s response across four metrics: safety, factual consistency, treatment appropriateness, and acknowledgement of patient inputs [14,54–57] (Table C in Appendix S2). The full prompt, including evaluator instructions is provided in Section B in S2 Appendix.

#### Data collection and analysis.

For each of the 10 JIA cases, we collected (1) treatment recommendations from the LLM-DA and each rheumatologist, and (2) ratings for the four metrics from each rheumatologist and LLM-Judge. Ratings were on a 1–3 ordinal scale, where 1 indicated an unsafe response, 2 an acceptable but incomplete response, and 3 an excellent and comprehensive response.

For quality metrics, we calculated descriptive statistics (i.e., mean and percent acceptable ratings) and estimated inter-rater using percent agreement and Gwet’s AC1 (Table 6) [58]:

**Table 6:** Interpretation of Gwet’s AC1 scores [59].

AC1 Score	Interpretation
< 0	Poor
0.00 - 0.20	Slight
0.21 - 0.40	Fair
0.41 - 0.60	Moderate
0.61 - 0.80	Substantial
0.81 - 1.00	Almost Perfect

- Percent acceptable was defined as the number of ratings considered acceptable (i.e., a score of 2 or 3), normalized by the total number of ratings for each metric.
- Percent agreement represented the proportion of observations in which raters assigned the same acceptability category; ratings of 2 or 3 were treated as equivalent (acceptable) and distinct from a rating of 1 (not acceptable).
- Gwet’s AC1 was used to measure chance-corrected inter-rater reliability. Statistical significance was calculated using a Wald test.

Given that scores of 2 or 3 were defined as clinically acceptable in the evaluation rubric descriptions, analyses were intentionally focused on establishing a baseline threshold of clinical utility for this proof-of-concept feasibility study.

For treatment recommendations, we calculated percent agreement between LLM-DA, each rheumatologist, and real-world data, defined as the proportion of identical drug-class combinations per treatment plan across comparators.

### Evaluation: Perceived value of LLM-DA

#### Overview.

We used a convergent mixed-methods design with a qualitative core [60]. Interview and focus group data were analyzed using a qualitative descriptive approach [61] and thematic analysis [62] to identify how an LLM-based decision aid influences adolescents’ collaboration with their JIA care team in treatment decisions. Three constructs guided the evaluation: (1) preparation for decision making, (2) collaboration dynamics during visit, and (3) perceived fit of the decision aid for JIA care.

Each session began with a brief demonstration of the decision aid, followed by a 30–45-minute discussion guided by a semi-structured interview guide (Section A in S3 Appendix) led by the study coordinator. To mitigate potential social desirability bias, we emphasized that there were no right or wrong answers and encouraged honest feedback, collected survey responses anonymously, and used neutral, non-leading language in the interview guide. Study details are presented in accordance with the consolidated criteria for reporting qualitative research (COREQ) guidelines [63] (Table A in S3 Appendix). Participants also completed two Ottawa Decision Support Framework questionnaires: (1) Acceptability and (2) Preparation for Decision Making (Sections B and C in S3 Appendix) [64]. Sessions took place in August 2025, either through in-person focus groups or online interviews.

Questionnaire data were summarized descriptively. Integration occurred at the analysis and interpretation stages using a weaving approach by embedding the questionnaire findings within theme write-ups.

#### Participant recruitment.

We recruited 27 individuals aged 12–18 through convenience sampling across four in-person focus groups. We also recruited seven individuals aged 19 or older for individual semi-structured online interviews using purposive sampling. Eligibility for both cohorts included an arthritis diagnosis, a past or current prescription of at least one arthritis medication, and English proficiency.

#### Data collection and analysis.

Qualitative data saturation was achieved through iterative analysis and triangulation of interview transcripts, field notes, and memoing [62,65]. We performed two cycles of coding. First, focus group transcripts were coded inductively and organized under the three pre-identified constructs that framed the evaluation approach. Using this codebook, we conducted a deductive analysis of the interview transcripts. Interviews were audio-recorded, transcribed, and analyzed using NVivo software (Version 15) by two researchers (AB and CL). Quantitative data gathered from two questionnaires were analyzed using descriptive statistics.

## Conclusion

This feasibility study provides preliminary evidence that an LLM-based decision aid may support collaborative decision-making in JIA by helping patients prepare for clinical encounters, reflect on treatment preferences, and engage more confidently with their care team. Rheumatologists rated most outputs as acceptable for safety and acknowledgment of patient inputs, supporting the potential value of the LLM-DA as a physician-mediated discussion aid. However, more modest ratings for factual consistency and treatment appropriateness, low treatment recommendation agreement, and variable inter-rater agreement indicate that the current system should not be interpreted as clinically ready for autonomous treatment recommendation. These findings highlight both the complexity of JIA treatment decision-making and the need for more rigorous evaluation methods that can distinguish acceptable clinical variation from model error. Future work should evaluate the LLM-DA using larger and more diverse case sets, consensus-based expert adjudication, prospective clinical workflows, and clearer rubrics that better capture factual consistency, treatment appropriateness, and preference-sensitive clinical reasoning.

## Supporting information

S1 Appendix Table A. Tripod-LLM Checklist for LLM evaluation. Generated using the interactive checklist available from the TRIPOD Group. The TRIPOD-LLM checklist is licensed under the Creative Commons Attribution 4.0 International License (CC BY 4.0). Retrieved from: https://tripod-llm.vercel.app/.
(DOCX)

S2 Appendix Table A. Patient preferences questionnaire. **Table B.** Patient clinical data collected, to be filled out manually or auto-populated from UCAN database. **Table C.** Each metric, with associated definition and scoring descriptions [74,75].
(DOCX)

S3 Appendix Figure A. ODSF Acceptability questionnaire results (Part 1). **Table A.** ODSF Acceptability questionnaire results (Part 2). **Figure B.** Preparation for Decision Making questionnaire.
(DOCX)

S4 Appendix Table A. Participant characteristics from both the focus group and interview sessions.
(DOCX)
