# Supplementary material for: An LLM-based decision aid for collaborative decision-making in juvenile idiopathic arthritis: A multimethod evaluation
Source: PLOS Digit Health. 2026 Oct 22;5(10):e0001655. doi: 10.1371/journal.pdig.0001655 (PMC13690242; doi:10.1371/journal.pdig.0001655)
Supplement: Supplementary file 3 [file pdig.0001655.s003.docx]

# S3 Appendix

**Section A: Data Collection Research Plan**

**Recruitment Announcement:**

*Hi everyone, my name is Antonia (she/her) and I am a PhD Candidate at the University of Toronto studying digital health. I am also a trainee at the Centre for Digital Therapeutics at the University Health Network located in Toronto, Ontario. I am conducting a study for my thesis project, which is focused on improving collaboration for JIA treatment decision-making. We developed a new tool that we think will help with this and we want to hear your honest feedback. If you are interested in participating, please sign up for an information session where I will share more study details.*

**Data collected during sessions:**

1. Qualitative data:
   a. Qualitative descriptive/thematic analysis from interview data to better understand if and how the decision aid would help facilitate collaboration
2. Quantitative data (questionnaires):
   a. Acceptability
   b. Preparation for decision-making
   c. Comfort with technology
   d. Patient demographic form

**Onboarding:**

1. Information Session: consent form discussion

*Welcome, and thank you for expressing interest to participate in this interview about a decision aid to help with making decisions for JIA treatments. My name is Antonia and I am the study coordinator for this project, and I will be conducting the interviews. I am also part of the development team who created the decision aid, but it’s important for you to remember that we are looking for your open and honest feedback. This includes sharing things that you like and do not like about the decision aid. There are no wrong answers, and the more feedback you can provide about the tool the more helpful it is for our research team.*

*Please let us know if you have questions at any time, and if you need a break or would like to stop the interview just let us know. You do not have to answer any questions that you do not want to. This part of the interview session will also be audio-recorded and is required for this study. If you do not want to be audio-recorded, you cannot participate in the study. Also, as we go through the interview, we will be taking notes so that we can review them at a later time.*

**Note:** Informed consent will be obtained prior to the interview session with each participant. The list below showcases the questions that will be asked during the interview sessions, but may include additional, follow-up questions based on participant response and feedback from previous interviews. The language of the questions may be simplified for younger participants.

2. Participant sign-off
3. *Activity:* Review the decision aid prototype

**Interview Part A: preparation for decision making (pre-visit readiness)**

1. *Activity*: imagine you are going to visit your doctor in the hospital to find a new medication that will work better for you. What are the top 3 things or questions you want to tell your doctor?
   a. What part of the decision aid made you think of these questions?
2. Do you think this tool would help you get ready for your doctor’s visit? Why or why not? How?
   a. What part helps you to get ready the most: questions about you, health info, or suggested medicines?
   b. How do you typically spend time before your doctor’s visit? Would anything change with this app?

**Interview Part B: Collaboration during visit (how the talk changes)**

1. What would you say to your doctor at your visit if you used this app? What is the first thing you would say to them?
   - (How would this change how you talk with your doctor and what you say?)
2. Point to one section or sentence in the results that you think is the most important for your doctor to see. Why?
3. Imagine that the doctor and this app gave different medicine suggestions. What would you do? How would you feel? What would you think?
   - Would you use the tool again? Would you still trust the results? Would this make you confused? Overwhelmed? Divert expertise to doctor?
   - Options: tell a parent, ask your doctor why, ignore the app, etc..

**Interview Part C: Acceptability and Appropriateness (perceived fit)**

1. How would this decision aid help you? Select one or share another way.
   - It would help me get ready for my visit
   - It would help me talk with my doctor at my visit
   - It would help me choose which medicine I would try next
   - Other:
2. Are the questions asked in the decision aid important to talk about with your doctor? Do you typically ask about these things? Why or why not?
3. Circle two “questions-about-you” that are the most important for your doctor to know**.** Why?
4. What is the most important thing in the decision aid’s output for you? Why?
5. Is there anything you think you wanted to see in the decision aid or something you usually think about or discuss with your doctor that ISN’T in the decision aid?
6. Would you tell a friend with JIA to use this tool? Why?
   a. What would you say to them about it?
7. Imagine you have an upcoming doctor’s visit to talk about your JIA. How would you want to use this tool? Why?
   - I want to fill it out before and for my doctor to see the results before my visit
   - I want to fill it out before, but I want to show it to my doctor myself during the visit
   - I want to fill it out during the visit and look at the results with my doctor
   - I want to use it by myself, but I don’t want to talk about it during the visit
   - I don’t want to use it
8. When would you want to use this app? 1) At home versus in the clinic, 2) alone or with your parents, 3) on what device?
9. Would you want to use this just when you have clinical visits or at other times too, to learn more about JIA?
10. Is there a certain age that you think this would be the most useful for you to use?
11. This decision aid uses large language models, like chat-gpt, to generate medication options. What are your initial thoughts about that?
    a. Would you trust the medications that are outputted?
    b. Does this fact impact what you would the decision aid for? For example, does this change what the most beneficial part of the tool is?

**Interview Part D: Usability (clarity of wording and ease of using this decision aid)**

1. Were the **questions and options** easy to understand?
   a. Are there any words or sentences that are unclear or confusing? Show me.
2. Were the **results** easy to understand?
   a. Are there any words or sentences that are unclear or confusing? Show me.

**Part E: (1) Acceptability and (2) Prep for DM Questionnaires**

Everyone to fill out separately but study coordinator to read each question out loud to ensure everyone understands the questions and fills it out

**Part F: (1) Patient Demographic and (2) Tech Comfort Level Questionnaires**

**Part G: Closing**

*Thank you very much for participating in this interview. Your time, insights, and feedback is invaluable to our research and will help us better ways to collaboratively manage JIA. We will analyze the information gathered from these interviews to inform the design of the decision aid. If you have any questions or need further information, please feel free to contact us. I will now stop the recording. Thank you again for your participation.*

*Table A: Reporting items from the CORE-Q Guidelines, with associated page numbers and comments, as applicable.*

| **No Item** | **Guide questions/description** | **Location in paper and/or Comments** |
| --- | --- | --- |
| **Domain 1: Research team and reflexivity - Personal Characteristics** | | |
| 1. Interviewer/facilitator | Which author/s conducted the interview or focus group? | Methods: Participant Recruitment |
| 2. Credential | What were the researcher’s credentials? E.g., PhD, MD | Appendix S5 |
| 3. Occupation | What was their occupation at the time of the study? | Appendix S5 |
| 4. Gender | Was the researcher male or female? | Appendix S5 |
| 5. Experience and training | What experience or training did the researcher have? | Appendix S5 |
| **Domain 1: Research team and reflexivity - Relationship with participants** | | |
| 6. Relationship established | Was a relationship established prior to study commencement? | No |
| 7. Participant knowledge of interviewer | What did the participants know about the researcher? e.g. personal goals, reasons for doing the research? | Appendix S5 |
| 8. Interviewer characteristics | What characteristics were reported about the interviewer/facilitator? e.g. Bias, assumptions, reasons and interests in the research topic | Appendix S5 |
| **Domain 2: Study design - Theoretical framework** | | |
| 9. Methodological orientation and Theory | What methodological orientation was stated to underpin the study? e.g. grounded theory, discourse analysis, ethnography, phenomenology, content analysis | Methods: Overview |
| **Domain 2: Study design - Participant selection** | | |
| 10. Sampling | How were participants selected? e.g., purposive, convenience, consecutive, snowball | Methods: Participant Recruitment |
| 11. Method of approach | How were participants approached? e.g., face-to-face, telephone, mail, email | Methods: Participant Recruitment |
| 12. Sample size | How many participants were in the study? | Methods: Participant Recruitment |
| 13. Non-participation Setting | How many people refused to participate or dropped out? Reasons? | No participants dropped out. |
| 14. Setting of data collection | Where was the data collected? e.g., home, clinic, workplace | Methods: Participant Recruitment |
| 15. Presence of nonparticipants | Was anyone else present besides the participants and researchers? | No |
| 16. Description of sample | What are the important characteristics of the sample? e.g. demographic data, date | Appendix S4 |
| **Domain 2: Study design - Data collection** | | |
| 17. Interview guide | Were questions, prompts, and guides provided by the authors? Was it pilot tested? | Methods: Data Collection and Analysis; Appendix S3. Questions were adjusted as needed through data collection. |
| 18. Repeat interviews | Were repeat interviews carried out? If yes, how many? | No |
| 19. Audio/visual recording | Did the research use audio or visual recording to collect the data? | Methods: Data Collection and Analysis |
| 20. Field notes | Were field notes made during and/or after the interview or focus group? | Methods: Data Collection and Analysis |
| 21. Duration | What was the duration of the interviews or focus group? | Methods: Overview |
| 22. Data saturation | Was data saturation discussed? | Methods: Data Collection and Analysis |
| 23. Transcripts returned | Were transcripts returned to participants for comment and/or correction? | No, but an effort was made during the interviews to clarify and confirm feedback. |
| **Domain 3: Analysis and Findings - Data Analysis** | | |
| 24. Number of data coders | How many data coders coded the data? | Methods: Data Collection and Analysis |
| 25. Description of the coding tree | Did the authors provide a description of the coding tree? | Methods: Data Collection and Analysis |
| 26. Derivation of themes | Were themes identified in advance or derived from the data? | Methods: Data Collection and Analysis |
| 27. Software | What software, if applicable, was used to manage the data? | Methods: Data Collection and Analysis |
| 28. Participant checking | Did participants provide feedback on the findings? | No, but an effort was made during the interviews to clarify and confirm feedback. |
| **Domain 3: Analysis and Findings - Reporting** | | |
| 29. Quotations presented | Were participant quotations presented to illustrate the themes/findings? Was each quotation identified? e.g., participant number | Results |
| 30. Data and findings consistent | Was there consistency between the data presented and the findings? | Results |
| 31. Clarity of major themes | Were major themes clearly presented in the findings? | Results |
| 32. Clarity of minor themes | Is there a description of diverse cases or a discussion of minor themes? | No minor themes identified. |

## Section B: Acceptability Questionnaire

We want to know what you think about the decision aid we just showed you!

1)	Rate each part of the app by picking one of the boxes for each page:

| Page | 😞 Not good | 😐 OK | 🙂 Good | 😃 Great | Not sure |
| --- | --- | --- | --- | --- | --- |
| Questions asking me about my preferences | ☐ | ☐ | ☐ | ☐ | ☐ |
| Medical history about my JIA | ☐ | ☐ | ☐ | ☐ | ☐ |
| Medicine options | ☐ | ☐ | ☐ | ☐ | ☐ |
| Information about each medicine | ☐ | ☐ | ☐ | ☐ | ☐ |
| Explanation for why the medications were chosen | ☐ | ☐ | ☐ | ☐ | ☐ |

2)	Do you think that the app is … (pick one):

☐ Too long 😐

☐ Too short 😐

☐ Just right 😃

☐ Not sure 🤔

3)	Do you think the screen with medicine suggestions (last page) had … (pick one):

☐ Too much information 😐

☐ Too little information 😐

☐ The right amount of information 😃

☐ Not sure 🤔

4)	The app (pick one) …

☐ Asked me too much stuff about my preferences 😐

☐ Asked me too much stuff about my medical history 😐

☐ Had a good mix asking me about my preferences and my medical history 😃

☐ Not sure 🤔

5)	The screen with medicine suggestions (last page) …

☐ Didn’t say a lot about my preferences 😐

☐ Didn’t say a lot about my medical history 😐

☐ Had a good mix of both my preferences and medical history 😃

☐ Not sure 🤔

6)	Would this app help you choose a medicine for your JIA?

☐ No 😕

☐ Maybe 😐

☐ Yes 😃

☐ Not sure 🤔

7)	Why do you think that?

___________________________________________________________________

___________________________________________________________________

8)	Would this app make choosing a medicine …

☐ Harder 😕

☐ About the same 😐

☐ Easier 😃

☐ Not sure 🤔

9)	Why do you think that?

___________________________________________________________________

___________________________________________________________________

10)	Do you think that this app is helpful?

☐ No 😐

☐ Yes 😃

☐ Not sure 🤔

11)	Why do you think that?

___________________________________________________________________

___________________________________________________________________

12)	Would this app help you talk with your doctor about medicines?

☐ No 😕

☐ Maybe 😐

☐ Yes 😃

☐ Not sure 🤔

13)	Why do you think that?

___________________________________________________________________

___________________________________________________________________

14)	What did you like the best about the decision aid?

___________________________________________________________________

___________________________________________________________________

15)	How can we make the decision aid better? What should we change or add?

___________________________________________________________________

___________________________________________________________________

## Section C: Preparation for Decision Making Questionnaire

| Think about your next visit. Imagine you used this tool before the visit. How much do you think it would… | 1 😞 Not at all | 2 😕 A little | 3 😐 Somewhat | 4 🙂 Quite a bit | 5 🤩 A lot! |
| --- | --- | --- | --- | --- | --- |
| 1. help me get ready to choose what to do next? | ☐ | ☐ | ☐ | ☐ | ☐ |
| 2. help me see the good and not-so-good parts of each option? | ☐ | ☐ | ☐ | ☐ | ☐ |
| 3. help me pick which of those good/not-so-good parts matter most to me? | ☐ | ☐ | ☐ | ☐ | ☐ |
| 4. help me see that my choice depends on both my health info and what matters to me? | ☐ | ☐ | ☐ | ☐ | ☐ |
| 5. help me figure out what I think for this decision? | ☐ | ☐ | ☐ | ☐ | ☐ |
| 6. help me decide how much I want to be involved in the decision? | ☐ | ☐ | ☐ | ☐ | ☐ |
| 7. help me write down questions to ask my doctor? | ☐ | ☐ | ☐ | ☐ | ☐ |
| 8. help me feel ready to tell my doctor what I think is most important? | ☐ | ☐ | ☐ | ☐ | ☐ |
| 9. help my doctor and me work together better? | ☐ | ☐ | ☐ | ☐ | ☐ |
| 10. help us use our visit time better? | ☐ | ☐ | ☐ | ☐ | ☐ |
| 11. make the visit go more smoothly? | ☐ | ☐ | ☐ | ☐ | ☐ |

*Figure A: ODSF Acceptability questionnaire results (Part 1)*

*Table B. ODSF Acceptability questionnaire results (Part 2)*

| **Questions** | **Answers** | | | |
| --- | --- | --- | --- | --- |
| Is the DA… | Too long  (0%) | Too short  (21%) | **Just right**  **(61%)** | Not sure  (18%) |
| The treatment recommendations had .. | Too much info  (12%) | Too little info  (21%) | **Good amount of info**  **(55%)** | Not sure  (12%) |
| The DA had... | Too much on preferences  (3%) | Too much on medical history  (6%) | **Good amount of both**  **(78%)** | Not sure  (13%) |
| The treatment recommendations had .. | Little preference info  (3%) | Little medical history info  (12%) | **Good amount of both**  **(48%)** | Not sure  (36%) |
| Would the DA help you choose a medicine? | No  (3%) | Maybe  (52%) | **Yes**  **(45%)** | N/A |
| The DA makes choosing medicine... | Harder  (3%) | The same  (21%) | **Easier**  **(67%)** | Not sure  (9%) |
| Is the DA helpful? | No  (3%) | N/A | **Yes**  **(82%)** | Not sure  (15%) |
| Would the DA help you talk to your doctor? | No  (21%) | Maybe  (30%) | **Yes**  **(48%)** | N/A |

*Figure B: Preparation for Decision Making questionnaire*
