# Supplementary material for: An LLM-based decision aid for collaborative decision-making in juvenile idiopathic arthritis: A multimethod evaluation
Source: PLOS Digit Health. 2026 Oct 22;5(10):e0001655. doi: 10.1371/journal.pdig.0001655 (PMC13690242; doi:10.1371/journal.pdig.0001655)
Supplement: Supplementary file 4 [file pdig.0001655.s004.docx]

# S4 Appendix - Participant Characteristics

*Table A. Participant characteristics from both the focus group and interview sessions*

| **Characteristic** | **Focus groups (N=27)** | **Interviews (N=7)** | **Total (N=34)** |
| --- | --- | --- | --- |
| Age (years) | Mean = 14.1  (SD = 1.9)  Range 12 to 18 | Mean = 22.2  (SD 1.9)  Range 19 to 23 | Mean = 15.6  (SD 3.7)  Range 12 to 23 |
| Gender identity | Girl (26); Boy (1); Missing (0) | Girl (4); Boy (2); Missing (1) | Girl (30); Boy (3); Missing (1) |
| Race/ethnicity |  |  |  |
| African/Black | 2 | 3 | 5 |
| Indo-Caribbean, Indo-African, Indo-Fijian, West-Indian | 1 | 0 | 1 |
| South Asian | 3 | 0 | 3 |
| Latin, South, or Central American | 3 | 0 | 3 |
| European/White | 15 | 4 | 19 |
| Indigenous within Canada | 4 | 0 | 4 |
| Preferred not to answer | 2 | 0 | 2 |
| Missing/not reported | 1 | 0 | 1 |
| JIA Sub-type |  |  |  |
| Enthesitis-related | 1 | 0 | 1 |
| Oligoarthritis | 1 | 2 | 3 |
| Psoriatic arthritis | 1 | 0 | 1 |
| Polyarthritis | 15 | 4 | 19 |
| Preferred not to answer | 1 | 0 | 1 |
| Unsure | 6 | 0 | 6 |
| Other | 1 | 0 | 1 |
| Missing/not reported | 1 | 1 | 2 |
| Time since diagnosis [years] | Mean = 7.6  (SD = 3.1); n=24 | Mean = 7.5  (SD = 6.1); n=5 | Mean = 7.6  (SD = 3.7); n=29 |
| Uveitis diagnosis | Yes (3); No (19); Unsure (5); Missing (0) | Yes (0); No (5); Unsure (0); Missing (2) | Yes (3); No (24); Unsure (5); Missing (2) |
| Current JIA discomfort [1-10 scale] | Mean = 5.0 (SD = 2.4); n=25 | Mean = 6.0 (SD = 1.6); n=5 | Mean = 5.2  (SD = 2.3); n=30 |
| Current medications [#] | Mean = 1.8  (SD = 1.6); n=25 | Mean = 2.0  (SD = 0.7); n=5 | Mean = 1.8  (SD = 1.5); n=30 |
| Previously prescribed and discontinued medications [#] | Mean = 3.5  (SD = 2.4); n=22 | Mean = 6.0  (SD = 5.3); n=5 | Mean = 4.0  (SD = 3.1); n=27 |
| Ever used Chat-GPT before? | Yes (21); No (6); Missing (0) | Yes (5); No (1); Missing (1) | Yes (26); No (7); Missing (1) |
| How often do you use Chat-GPT? |  |  |  |
| Every day | 2 | 2 | 4 |
| A few times in a week | 11 | 1 | 12 |
| A few times in a month | 4 | 1 | 5 |
| Every few months | 4 | 1 | 5 |
| Missing/not reported | 0 | 1 | 1 |
| What do you use Chat-GPT for? |  |  |  |
| School and homework | 14 | 4 | 18 |
| Fun | 9 | 2 | 11 |
| To look up health information | 6 | 4 | 10 |
| Other | 3 | 1 | 4 |
| Missing/not reported | 7 | 1 | 8 |
| How much do you trust Chat-GPT? |  |  |  |
| I usually trust everything it says | 6 | 0 | 6 |
| I trust a little bit of what it says | 14 | 3 | 17 |
| I don’t trust anything it says | 1 | 1 | 2 |
| Missing/not reported | 7 | 2 | 9 |

* Sex assigned at birth mirrored gender identity for all participants except one
